# Genetic variant of *ADH1C* for predicting survival in esophageal squamous cell cancer patients who underwent postoperative radiotherapy

**DOI:** 10.3389/fgene.2022.988433

**Published:** 2022-09-21

**Authors:** Xin Xu, Zhiyong Sun, Ling Rong, Xiaohang Wang, Lei Xu, Jun Lu, Qing Ye, Lei Zhang, Yongrui Bai, Xiumei Ma

**Affiliations:** ^1^ Department of Radiation Oncology, Renji Hospital, School of Medicine, Shanghai Jiao Tong University, Shanghai, China; ^2^ Department of Thoracic Surgery, Renji Hospital, School of Medicine, Shanghai Jiao Tong University, Shanghai, China; ^3^ Department of Research, Medical Laboratory of Nantong Zhongke, Nantong, China

**Keywords:** esophageal squamous cell cancer, adjuvant radiotherapy, ADH1C, nomogram, rs1789924

## Abstract

**Background:** Single nucleotide polymorphisms (SNPs) of essential enzymes for alcohol metabolism ADH1B, ADH1C, and ALDH2 are commonly regarded as genetic biomarkers for esophageal squamous cell carcinoma (ESCC) susceptibility. However, there have not been any reports on relations between SNPs of these genes and the prognosis of postoperative radiotherapy in ESCC. The current study aimed to understand the associations between gene variants of alcohol metabolism and adjuvant radiotherapy’s prognosis in ESCC.

**Methods:** This study retrospectively analyzed 110 ESCC patients from our institution who received adjuvant radiotherapy after surgery. The SNPs of ADH1B rs1229984, ADH1C rs1789924, and ALDH2 rs671 were detected by Sanger sequencing using formalin-fixed paraffin-embedded tumor samples. A nomogram was drawn based on prognostic factors associated with overall survival (OS).

**Results:** ADH1C rs1789924 (C>T) was associated with poor DFS and OS in ESCC patients undergoing adjuvant radiotherapy. Multivariate analysis showed that ADH1C rs1789924 (C>T) was one of the independent prognosis factors of DFS and OS. However, the genotypes of ADH1B SNP rs1229984 and ALDH2 rs671 were not associated with differences in the PFS and OS of these patients. Compared with the AJCC staging system, the nomogram containing the ADH1C genotype can more effectively and accurately predict the survival time of ESCC after surgery and adjuvant radiotherapy.

**Conclusion:** ADH1C rs1789924 might be a prognostic genetic biomarker for ESCC patients undergoing surgery and postoperative radiotherapy.

## 1 Introduction

Esophageal cancer is known as the eighth most common cancer worldwide and is the sixth leading cause of cancer-related deaths (Sung et al., 2021). Domestically, it is the fifth most common cancer and the fourth most common cause of cancer death, with approximately 346,633 new cases and 323,600 deaths in 2022 (Xia et al., 2022). China is one of the countries showing the highest incidence rate of ESCC, a primary histological type of esophageal cancer (Smyth et al., 2017). Alcohol consumption, smoking, poor nutrition, and some dietary factors like consumption of very hot beverages are considered risk factors for ESCC. Genetic factors may also play a vital role in susceptibility to ESCC (Smyth et al., 2017). A controlled study of Chinese patients concluded that single nucleotide polymorphisms (SNPs) of important enzymes for alcohol metabolism ADH1B, ADH1C, and ALDH2 are commonly regarded as genetic biomarkers of ESCC susceptibility (Gao et al., 2013). In Liu’s study, ALDH2 rs671 affected not only the susceptibility to ESCC but also its poor prognosis (Liu et al., 2018). The *ADH* gene was also associated with the prognosis of some other solid tumors (Wang et al., 2018; Shen et al., 2019; Chen et al., 2020; Liu et al., 2020) and response to chemotherapy (Khrunin et al., 2014; Le Morvan et al., 2015). However, there have not been any reports on relations between SNPs of these genes and the prognosis of postoperative radiotherapy in ESCC.

For resectable local advanced esophageal cancer, although neoadjuvant chemoradiotherapy followed by surgery was recommended, the vast majority of patients in China initially choose surgery. Relevant studies have shown that treatment failure is mainly due to local recurrence. Therefore, postoperative adjuvant therapy has a significant effect. According to Xiao’s research conclusion, postoperative radiotherapy can effectively reduce the probability of locoregional recurrence for all patients and can improve the survival of stage III or positive lymph node metastatic esophageal carcinoma (Xiao et al., 2003; Xiao et al., 2005). A randomized controlled trial of phase III suggested that postoperative radiotherapy, especially postoperative chemoradiotherapy, significantly improved DFS and OS in stage IIB–III esophageal squamous cell carcinoma (Ni et al., 2021). Even for patients with relatively early-stage T2–3N0M0, it was also well documented that postoperative radiotherapy significantly increased the patients’ DFS and reduced the likelihood of the local regional recurrence rate (Deng et al., 2020). Moreover, significant variability in disease response is observed for patients who underwent adjuvant radiotherapy. Several studies have demonstrated a correlation between clinical factors and prognosis. However, because such individual differences are difficult to predict precisely, biomarkers must be identified to screen patients for whom adjuvant therapy is not beneficial. Therefore, the purpose of our study is to more comprehensively and accurately understand the correlation between the variations of alcohol metabolism genes and the prognosis of adjuvant radiotherapy in ESCC.

## 2 Materials and methods

### 2.1 Patients

By sorting out and summarizing the relevant data on ESCC patients who were selected for postoperative radiotherapy in the Radiation Oncology Department of Renji Hospital from April 2008 to October 2018, 110 patients were retrospectively analyzed. The main inclusion criteria were as follows: 1) the age of the patients must be between 18 and 80 years; 2) according to the eighth edition staging system promulgated by the American Joint Committee on Cancer (AJCC), the patients must meet the diagnostic criteria for stage II–IVa thoracic esophageal squamous cell carcinoma; 3) the overall condition of the patients must be good, that is, the Eastern Cooperative Oncology Group performance status of 0 or 1; 4) there should be no abnormality in liver, kidney, and bone marrow functions; 5) all the patients should have undergone radical surgery in the Department of Thoracic Surgery of Renji hospital and received adjuvant radiotherapy in 3 months after surgery; 6) formalin-fixed, paraffin-embedded tumor tissue of the patients should be available; and 7) the patients should be under a regular follow-up after treatment. The exclusion criteria were as follows: 1) Patients with palliative resection and tumor residual; 2) tumor tissue should be unavailable; 3) radiation dose should be less than 40 Gy; 4) loss of follow-up after treatment; and 5) concurrent malignancy or previous malignancy within the past 5 years. This study was approved by the ethics committee of Renji Hospital.

### 2.2 Treatment

#### 2.2.1 Surgery

All patients underwent esophagectomy and lymph node dissection. The surgical plan was chosen according to the different locations of the tumor. For example, esophageal cancers in the upper and middle thoracic segments were generally suitable for Ivor Lewis or McKeown surgery, while those located in the lower thoracic segment were more suitable for Sweet esophagectomy. All patients were R0 resectioned.

#### 2.2.2 Adjuvant radiotherapy

The optimal time to receive adjuvant radiation therapy is 4–12 weeks after surgery. All patients before 2011 were treated with three-dimensional conformal radiotherapy; after 2011, most of the patients received intensity-modulated radiotherapy (IMRT). The median radiation dose was 50 Gy, ranging from 40 to 60 Gy in 20–30 fractions (2 Gy per fraction). The clinical target volume (CTV) was determined by the location of the primary tumor and the positive nodes found during pathological examination or surgery. For upper thoracic tumors, the boundary of the CTV was at the superior border of the cricothyroid membrane, whereas for midthoracic tumors, the border was at the superior border of the first thoracic vertebra. The lower border was located 3.0 cm below the carina and may also be at the lower border of the tumor bed, combined with the location of the tumor. The CTV includes the bilateral supraclavicular region and mediastinal stations 2R/L, 4R/L, 7, and 8, according to the tumor location. The planning target volume (PTV) was formed by a uniform 0.5 cm expansion around the CTV. Chemotherapy (sequential or concurrent with radiotherapy) was given if necessary.

### 2.3 Follow-up

Follow-up is required after the treatment. The frequency of follow-up is quarterly for the first 2 years after surgery, semi-annually for the second 2 years, and can be extended to yearly thereafter. Diagnostic imaging and endoscopic biopsy are mainly used to check for esophageal recurrence. Enhanced CT, MRI, or PET-CT is used to check whether there is local recurrence and distant metastasis, and fine needle aspiration is also required if necessary.

### 2.4 Genotyping assays

DNA was extracted from paraffin block sections of tumor samples during surgery with the aid of Qiagen kits. The SNPs of ADH1B rs1229984, ADH1C rs1789924, and ALDH2 rs671 were detected by Sanger sequencing. The primers used for PCR are listed as follow: rs1229984-F: 5′-CTT​TCG​TCT​CTC​ATT​GCC​T-3′, rs1229984-R: 5′-TAA​CCT​TGG​GGA​TAA​ACT​GA-3’; rs1789924-F: 5′-TAA​AGA​AAT​GGG​CAC​CGA-3′, rs1789924-R 5′-CCC​CTT​TGC​TGT​GAC​TGA-3’; and rs671-F: 5′-CCC​ATA​ACC​CCC​AAG​AGT-3′, rs671-R: 5′-CAG​AGC​AGA​GGC​TGG​GTC-3’. The PCR product was sequenced on an ABI 3100 DNA analyzer (Applied Biosystems, Foster City, CA, United States), and the data were analyzed by Sequencer 4.9 software.

### 2.5 Statistical analyses

Further statistical analyses were carried out with the help of SPSS 22.0 software (SPSS Inc., Chicago, IL, United States). Categorical variables were compared using Pearson’s chi-squared or Fisher’s exact tests. Survival analysis was performed by the Kaplan–Meier method, followed by log-rank tests. Univariable and multivariable Cox regression analyses were then used to identify risk factors for disease-free survival (DFS) and overall survival (OS). OS time was the interval from the date of surgery to death or the most recent follow-up time, which was 31 December 2021. DFS is defined as survival time without disease progression from the date of surgery. All *p*-values were two-sided; values of *p* < 0.05 were considered statistically significant. R 3.4.4 software (Institute for Statistics and Mathematics, Vienna, Austria) was used to draw a nomogram of potential prognostic factors significantly associated with OS, and the calibration curve and the concordance index (C-index) were used to judge its accuracy.

## 3 Results

### 3.1 Patient characteristics

In the study, the relevant data on 110 ESCC patients who received adjuvant radiotherapy were selected as the research sample. The clinical characteristics of these patients are detailed in Table 1. The selected patients ranged in age from 44 to 80 years, with an average age of 61 years. In the overall sample, 75 patients had stage III, 31 had stage II, and 4 had stage IVa disease. Only 78 patients were evaluable for rs1229984 genotypes, including 38 patients with genotype TT, 35 patients with genotype TC, and 5 patients with genotype CC. All 110 patients were evaluated for rs1229984 SNP status, including 100 patients with wild-type CC and 10 patients with variant-type CT; 100 patients were evaluable for rs671 genotypes, including 49 patients with genotype GG, 48 with genotype GA, and 3 with genotype AA.

**TABLE 1:** Clinical features and genotypes of all the patients.

Characteristic	Total N = 110 (%)
Age (years)	
≥65	35 (31.8)
<65	75 (68.2)
Gender	
Male	90 (81.8)
Female	20 (18.2)
Location	
Upper	18 (16.4)
Middle	50 (45.5)
Lower	42 (38.2)
Length (cm)	
<5 cm	60 (54.5)
≥5 cm	50 (45.5)
pT stage	
T2	20 (18.2)
T3	66 (60.0)
T4	24 (21.8)
pN stage	
N0	39 (35.5)
N1	47 (42.7)
N2	18 (16.4)
N3	6 (5.5)
TNM stage	
II	31 (28.2)
III	75 (68.2)
IVa	4 (3.6)
Adjuvant chemotherapy	
Yes	60 (54.5)
No	50 (45.5)
rs1229984 genotypes	
TT	38 (34.5)
TC	35 (31.8)
CC	5 (4.5)
Unknown	32 (29.1)
rs1789924 genotypes	
CC	100 (90.9)
CT	10 (9.1)
rs671 genotypes	
GG	49 (44.5)
GA	48 (43.6)
AA	3 (2.7)
Unknown	10 (9.1)

### 3.2 Comparing DFS and OS of patients in different genotypes

**FIGURE 1:**
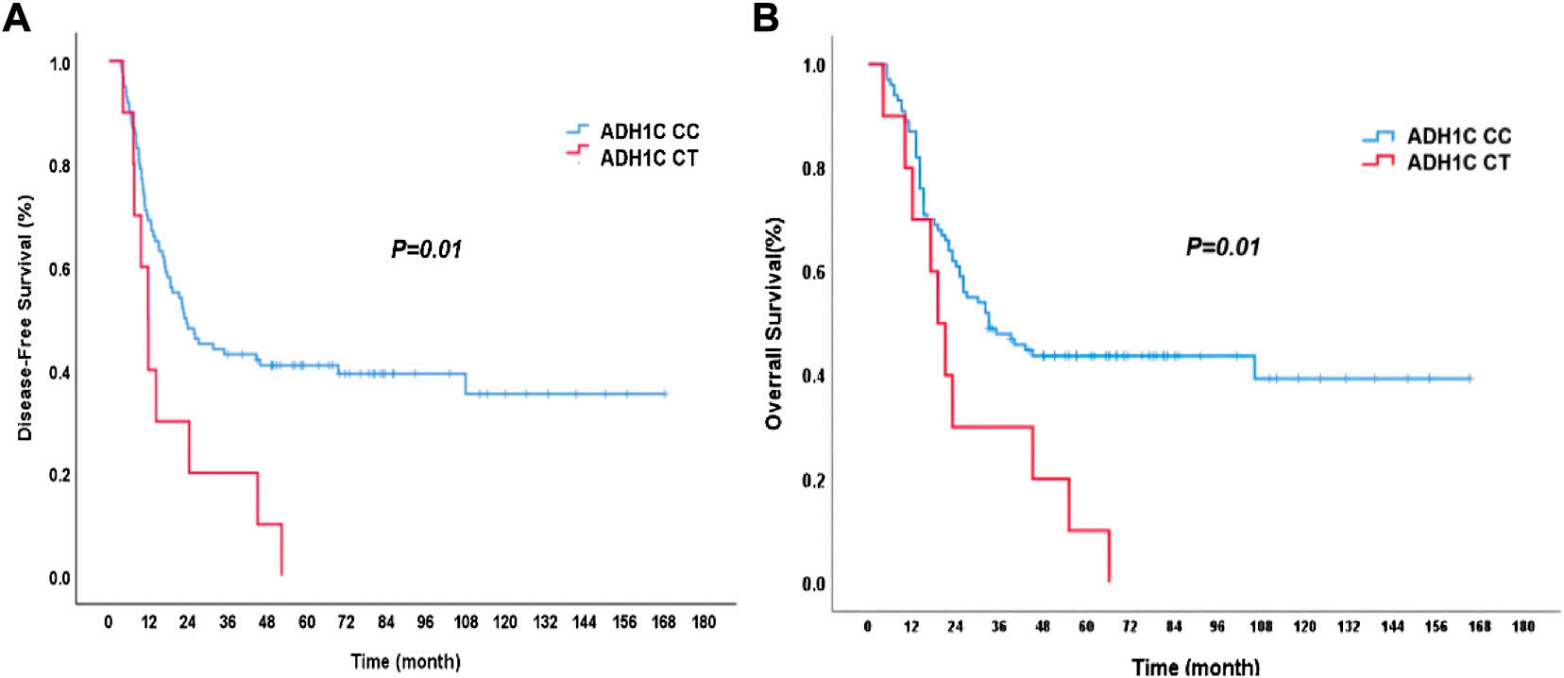
Disease-free survival **(A)** and overall survival **(B)** curves of the patients with different ADH1C rs1789924 genotypes. CC: wild type; CT: variant type.

During follow-up, 67 patients died and 69 patients were with disease progression. For the surviving patients, the median follow-up time was 70 months. The mean DFS was 22.2 months (95% CI: 15.2–29.1 months), and the mean OS was 32.0 months (95% CI: 20.3–43.7 months) for the whole group of patients. The genotypes of ADH1C SNP rs1789924 were significantly associated with differences in the PFS and OS. The patients with variant-type CT had much worse DFS and OS than those with wild-type CC (Figure 1). The mean DFS was 22.8 months in patients with rs1789924 CC type and 12.8 months in patients with rs1789924 CT type (*p* = 0.01), and the mean OS was 33.0 and 19.0 months, respectively (*p* = 0.01). However, the genotypes of ADH1B SNP rs1229984 and ALDH2 rs671 were not associated with differences in the PFS and OS of these patients. The clinical characteristics of different rs1229984 genotypes are shown in Supplementary Table S1 *.* There were no significant differences between the two groups in all clinical features except tumor length. More patients in the mutant group had tumors longer than 5 cm.

### 3.3 Univariate and multivariate analyses for DFS and OS

In the univariate analysis of clinical characteristics and genotypes, as shown in Table 2, TNM stage, N stage, tumor length, and ADH1C were significantly correlated with DFS. The variables with *p* < 0.2 in univariate analysis were subjected to multivariate analysis. In addition, independent associations between TNM stage, tumor length, and ADH1C and DFS were confirmed by multivariate analysis. In the analysis of the variables associated with the OS, age, TNM stage, N stage, tumor length, and ADH1C were significantly associated with OS. In addition, in multivariate analysis, age, TNM stage, tumor length, and ADH1C were independently associated with OS (Table 3). Therefore, we considered that ADH1C SNP rs1789924 might be one of the independent prognostic factors for ESCC patients who underwent surgery and adjuvant radiotherapy.

**TABLE 2:** Univariate analysis of clinical parameters and rs1789924 genotypes in predicting DFS.

		Univariate analysis			Multivariate analysis		
Parameter	Comparison	*p*-value	HR	95% CI	*p*-value	HR	95% CI
Age	<65 vs ≥65	0.150	1.423	0.880–2.303	0.057	1.609	0.986–2.625
Stage	II vs III & IVa	0.037*	1.811	1.035–3.170	0.030*	1.965	1.066–3.622
N stage	N0–1 vs N2–3	0.016*	1.915	1.127–3.253	0.079	1.664	0.943–2.936
Tumor length	<5 cm vs ≥5 cm	0.033*	1.663	1.041–2.655	0.031*	1.718	1.051–2.809
ADH1C	CC vs CT	0.011*	2.392	1.219–4.694	0.022*	2.289	1.125–4.658

*p < 0.05.

**TABLE 3:** Univariate analysis of clinical parameters and rs1789924 genotypes in predicting OS.

		Univariate analysis			Multivariate analysis		
Parameter	Comparison	*p*-value	HR	95% CI	*p*-value	HR	95% CI
Age	<65 vs ≥65	0.028*	1.732	1.060–2.829	0.008*	1.979	1.199–3.268
Stage	II vs. III & IVa	0.029*	1.934	1.070–3.494	0.024*	2.106	1.105–4.016
N stage	N0–1 vs. N2–3	0.011*	1.999	1.173–3.405	0.083	1.662	0.936–2.951
Tumor length	<5 cm vs. ≥5 cm	0.017*	1.798	1.109–2.915	0.018*	1.836	1.109–3.038
ADH1C	CC vs. CT	0.014*	2.339	1.191–4.595	0.03*	2.196	1.079–4.470

*p < 0.05.

### 3.4 Nomogram for predicting OS

**FIGURE2:**
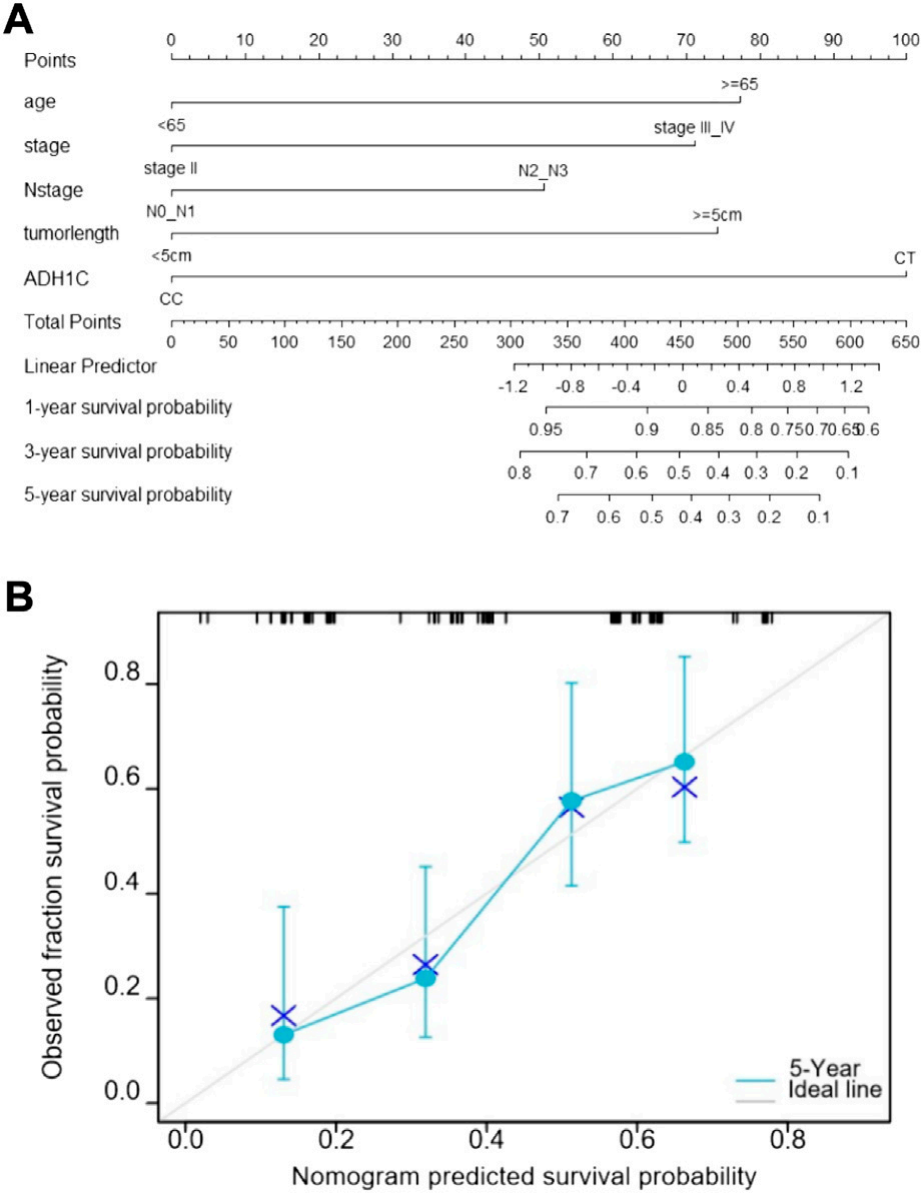
Prediction nomogram for overall survival. **(A)** Nomogram predicts OS based on age, TNM stage, N stage, tumor length, and ADH1C genotype. **(B)** Calibration curve of the nomogram.

**FIGURE3:**
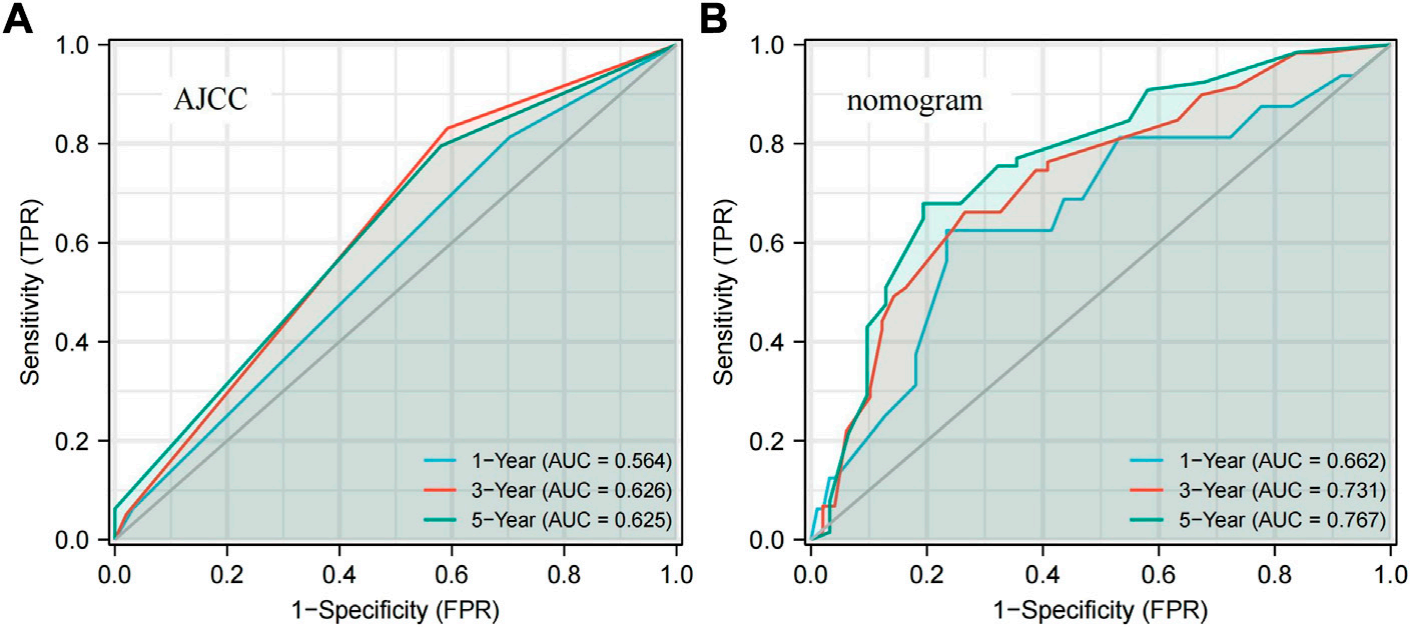
ROC curves present the predictive power for 1-year, 3-year, and 5-year OS. **(A)** AJCC stage. **(B)** Nomogram. ROC: receiver operator characteristic; AJCC: American Joint Committee on Cancer.

Based on the five prognostic factors screened in the multivariate Cox regression analysis, a nomogram was drawn for predicting 1-year, 3-year, and 5-year survival probabilities (Figure 2A). The 5-year OS probability calibration curve showed that the predicted values of the nomogram had a high agreement with the actual observed values of OS (Figure 2B). In addition, the calculated C-index result for the predicted nomogram was 0.662 (95% CI: 0.625–0.700). Afterward, the accuracy of the predicted results of the nomogram and the AJCC staging system was compared in detail with the help of ROC analysis. The calculation found that the AUC values of the OS of the predicted nomogram in the aforementioned three different periods were 0.662, 0.731, and 0.767, which were obviously higher than 0.564, 0.626, and 0.625 of the staging system. The nomogram has a better discriminative ability than the AJCC staging system (Figure 3). These results suggested that the nomogram, including the ADH1C genotype and other clinical characteristics, is better at predicting survival for ESCC after surgery and adjuvant radiotherapy.

## 4 Discussion

This study focuses on analyzing associations between SNPs of essential enzymes for alcohol metabolism ADH1B, ADH1C, and ALDH2 and survival in esophageal cancer patients receiving postoperative radiotherapy. We had shown a significant association between ADH1C rs1789924 genotypes with DFS and OS. The patients with variant-type rs1789924 had much worse DFS and OS than those with wild-type rs1789924.

Alcohol metabolism mainly depends on alcohol dehydrogenases (ADH), which oxidize ethanol to acetaldehyde or ketones (Zhang, Mai, and Huang 2010). *ADH1B* and *ADH1C* are the most common ADH genes and encode the most critical components of the ADH enzyme subunit. Alcohol metabolism also requires another enzyme, encoded by ALDH2. It has a high affinity to acetaldehyde and is able to facilitate the conversion of acetaldehyde to non-toxic acetate (Seitz and Stickel 2007). There are three SNPs, rs1229984 in ADH1B at 4q23, rs1789924 near ADH1C at 4q23, and rs671 in ALDH2 at 12q24, significantly associated with the risk of ESCC in the Chinese population (Gao et al., 2013). The SNP rs1229984 is a missense polymorphism (A>G, His48Arg) within the ADH1B gene, which encodes a more active ADH enzyme. The SNP rs1789924, located at 5′ near the gene region of *ADH1C*, may affect transcription factor binding. The SNP rs1789924 has a significant relationship with another SNP, rs698, at 4q23. The variant A allele of rs671 (G>A, Glu504Lys) was able to significantly reduce the metabolic activity of the ALDH2 enzyme for acetaldehyde (Yuan et al., 2013). Gao and other related scholars selected 2,139 ESCC cases and 2,273 control cases as samples and found that minor alleles of rs1229984 and rs1789924 could significantly increase the risk of ESCC. On the contrary, the minor allele of rs671 could significantly reduce its risk (Gao et al., 2013). This study explored the correlation between these genetic biomarkers for ESCC prognosis. We only found that the variant T allele of rs1789924 in *ADH1C* was associated with the prognosis of ESCC patients electing for surgery and receiving adjuvant radiotherapy.

Some analyses found that *ADH1C* plays a vital role in developing breast, liver, colorectal, and lung cancers. Some studies found that the expression of *ADH1C* was significantly downregulated in hepatocellular carcinoma tumor samples compared with normal liver samples and whose high expression of ADH1C was significantly associated with a good survival rate in liver cancer patients (Chen et al., 2020; Liu et al., 2020). It has also been concluded that with the continuous reduction of ADH1C expression levels, the prognosis of colorectal cancer patients can gradually worsen (Li et al., 2022). The conclusion of the study by Kumamoto et al., (2019) showed that *ADH1C* could also be used to predict the recurrence rate of stage III colorectal cancer patients after chemotherapy. For lung cancer patients, high expression of *ADH1B*, *ADH1C*, *ADH4*, and *ADH5* genes can achieve a better prognosis. In addition, the expression of ADH family members was associated with smoking status, clinical stage, and chemotherapy status (Wang et al., 2018). Feng’s study showed that the upregulated expression of *ADH1C* enhances cisplatin resistance of lung adenocarcinoma cells (Jiang et al., 2022). Some studies have shown the correlation between SNPs of ADH1C and cancer prognosis. The SNP rs698 in ADH1C significantly affects complete tumor response in ovarian cancer patients receiving cisplatin for chemotherapy (Khrunin et al., 2014). A randomized phase III trial found that another SNP rs1693482 in ADH1C significantly affected OS in breast cancer patients undergoing neoadjuvant chemotherapy and without the need to achieve PCR (Le Morvan et al., 2015). Through a study in Xinjiang Han and Kazakh populations in China, it was found that ALDH2 rs671 (G>A) is not only closely related to the susceptibility to ESCC in Kazak populations but also significantly correlated with poor prognosis of EC in both Kazak and Han ethnic groups (Liu et al., 2018).

It is understood that this study is the first to show that SNP rs1789924 near ADH1C significantly affected the DFS and OS of ESCC patients undergoing surgery and adjuvant radiotherapy. In our multivariate analysis, the rs1789924 genotype was the independent prognostic factor for both DFS and OS. In the multivariate analysis, we also demonstrated that some clinical characteristics were correlated with survival. For these patients, the TNM stage and tumor length were independently associated with PFS, and the TNM stage, tumor length, and age were independently associated with OS. This result was consistent with our previous study (Xu et al., 2016) and some other scholars’ studies (Zou et al., 2020). For ESCC patients undergoing surgery, the pathological stage is the most critical prognostic factor and the key basis for adjuvant therapy after surgery. Although postoperative adjuvant therapy is not recommended for patients with R0 resection according to the NCCN guidelines, for stage IIb–III patients, especially those with positive lymph nodes, adjuvant radiotherapy can effectively reduce the local relapse and improve survival. A phase III randomized controlled trial in China has demonstrated that postoperative treatment (PORT/POCRT) may significantly prolong the survival in these patients. The pathological TNM stage and treatment regimen can significantly affect the DFS and OS (Ni et al., 2021). In our study, the TNM stage was also found as an independent prognostic factor for OS and DFS, but the predictive capability for survival was poor. Therefore, it is necessary to explore a better predictive survival model that can provide counseling and treatment guidance services to patients. The nomogram (including the rs1789924 genotype) based on the multivariate analysis results during this study can be used to predict OS accurately in ESCC patients.

In our study, rs1789924 variant-type CT was identified only in 9.1% (10/110) of patients, which was lower than the frequency reported in the former research (Gao et al., 2013). This may be due to the small sample size of our research, which was the first limitation to the study. Second, this was a retrospective, single-center study that might limit the results’ universality. The result should be validated in a larger population in the future study. Third, as it was a retrospective study, we could not obtain information on the drinking history of all patients, so we did not concern about this aspect. Finally, the mechanism of action of ADH1C in cancer has not been clearly understood, and more extensive and in-depth mechanism studies are needed to better understand the role of rs1789924 in ESCC. Therefore, extensive sample collection and molecular mechanism studies are needed to expand the study to validate these preliminary results and explore the mechanisms of impact.

## 5 Conclusion

Our study first reported a significant association between ADH1C rs1789924 genotypes with DFS and OS for ESCC patients undergoing surgery and postoperative radiotherapy. The SNP of rs1789924 was an independent prognostic factor for these patients. The developed nomogram integrating clinical features and the rs1789924 genotype showed superior prediction ability for OS, which might help us develop individualized postoperative adjuvant therapy strategies.

## Data Availability

The original contributions presented in the study are included in the article/Supplementary Material; further inquiries can be directed to the corresponding authors.
