# Supplementary material for: Genetic variant of ADH1C for predicting survival in esophageal squamous cell cancer patients who underwent postoperative radiotherapy
Source: Front Genet. 2022 Sep 21;13:988433. doi: 10.3389/fgene.2022.988433 (PMC9532693; doi:10.3389/fgene.2022.988433)
Supplement: Supplementary file 1 [file Table1.docx]

| **TABLE S1∣**Clinical characteristics in patients with different ADH1C genotypes. | | | |
| --- | --- | --- | --- |
| **Characteristics** | **rs1789924 CC**  **N=100** | **rs1789924 CT**  **N=10** | **P value** |
| Age (years) |  |  | 0.63 |
| ≥65 | 33 (33.0) | 2 (20.0) |  |
| <65 | 67 (67.0) | 8 (80.1) |  |
| Gender |  |  | 0.26 |
| Male | 80 (80.0) | 10 (100.0) |  |
| Female | 20 (20.0) | 0 (0.0) |  |
| Location |  |  | 0.85 |
| Upper | 17 (17.0) | 1 (10.0) |  |
| Middle | 45 (45.0) | 5 (50.0) |  |
| Lower | 38 (38.0) | 4 (40.0) |  |
| Length (cm) |  |  | 0.00 |
| <5cm | 57 (57.0) | 3 (30.0) |  |
| ≥5cm | 43 (43.0) | 7 (70.0) |  |
| pT-stage |  |  | 0.08 |
| T2 | 19 (19.0) | 1 (10.0) |  |
| T3 | 62 (62.0) | 4 (40.0) |  |
| T4 | 19 (19.0) | 5 (50.0) |  |
| pN-stage |  |  | 0.86 |
| N0 | 35 (35.0) | 4 (40.0) |  |
| N1 | 43 (43.0) | 4 (40.0) |  |
| N2 | 16 (16.0) | 2 (16.0) |  |
| N3 | 6 (6.0) | 0 (0.0) |  |
| TNM-stage |  |  | 0.59 |
| II | 27 (27.0) | 4 (40.0) |  |
| III | 69 (69.0) | 6 (60.0) |  |
| IVa | 4 (4.0) | 0 (0.0) |  |
| Adjuvant chemotherapy |  |  | 0.53 |
| Yes | 56 (56.0) | 4 (40.0) |  |
| No | 44 (44.0) | 6 (60.0) |  |
